# Top-down determinants of the numerosity–time interaction

**DOI:** 10.1038/s41598-023-47507-9

**Published:** 2023-11-30

**Authors:** Irene Petrizzo, Michele Pellegrino, Giovanni Anobile, Fabrizio Doricchi, Roberto Arrighi

**Affiliations:** 1 Department of Neuroscience, Psychology, Pharmacology, and Child Health, University of Florence, 50139 Florence, Italy; 2 Dipartimento di Psicologia 39, Università degli Studi di Roma “La Sapienza”, Rome, Italy

**Keywords:** Perception, Sensory processing

## Abstract

Previous studies have reported that larger visual stimuli are perceived as lasting longer than smaller ones. However, this effect disappears when participants provide a qualitative judgment, by stating whether two stimuli have the “same or different” duration, instead of providing an explicit quantitative judgment (which stimulus lasts longer). Here, we extended these observations to the interaction between the numerosity of visual stimuli, i.e. clouds of dots, and their duration. With “longer vs shorter” responses, participants judged larger numerosities as lasting longer than smaller ones, both when the responses were related to the order (Experiment 1) or color (Experiment 4) of stimuli. In contrast, no similar effect was found with “same vs different” responses (Experiment 2) and in a time motor reproduction task (Experiment 3). The numerosity–time interference in Experiment 1 and Experiment 4 was not due to task difficulty, as sensory precision was equivalent to that of Experiment 2. We conclude that in humans the functional interaction between numerosity and time is not guided, in the main, by a shared bottom-up mechanism of magnitude coding. Rather, high-level and top-down processes involved in decision-making and guided by the use of “magnitude-related” response codes play a crucial role in triggering interference among different magnitude domains.

## Introduction

Recently, much research has been dedicated to investigating whether the human brain encodes different perceptual dimensions such as duration, size or numerosity via single or via multiple and functionally independent magnitude representations. Indeed, it has been pointed out that the brain frequently needs to process quantitative inputs coming from different dimensions such as space, time and number at the same time, and these magnitudes often correlate with each other. Just as the longer the distance to walk, the longer the time needed to reach the destination and the more steps to get there. The ATOM Theory^1^ proposes that a shared neural and functional mechanism of magnitude representation might be an efficient interface to combine perceptual information with the programming and execution of the motor routines needed to interact with objects in the environment.

Indeed, several studies have reported similarities between the perception of space, time, and number. For example, the discrimination of temporal, numerical and spatial magnitude follows the same psychophysical law—Weber’s law—with the just noticeable difference (JND) between two stimuli being proportional to the overall intensity level^2–4^. Furthermore, the distance effect reported in numerical judgements, according to which the higher the numerical distance between two numbers, the easier to discriminate between them, also occurs for quantitative judgements of other dimensions such as length^5,6^ and duration^7^. Other similarities have been reported regarding the effects of contextual information. After observing a fast-moving visual stimulus for a few seconds (motion adaptation), the perceived time^8,9^, numerosity^10^ and apparent position^11^ are all robustly compressed. Similarly, eye movements have been reported to distort perceived time, space, and numerosity with a strong compression of all these dimensions at the time of the saccadic onset^12,13^. Recently, it has been shown that the interaction between action and perception also occurs for other effectors, as the repetitive execution of hand routines (motor adaptation) compresses or extends the perceived time, space, and numerosity depending on the number of movements executed^14–18^.

A possible consequence of a shared processing might be the existence of interference effects amongst them when they get presented together, even when the information from a given dimension is not relevant to accomplish the task. For example, observers that must select the more numerous between two visual arrays of dots in which the number of dots and the area occupied by dots are congruent (i.e. the more numerous array also has a greater area), or incongruent (i.e. area is kept constant so that the more numerous array has smaller individual elements than the less numerous one), are slower and less accurate in the incongruent trials^19^.

The interplay between spatial and temporal information processing is also prone to cross-interference. Several studies reported that larger stimuli are perceived as lasting longer than smaller ones^20–22^. However, while irrelevant spatial information cannot be ignored when making judgments about stimuli duration, the opposite is not true as the performance of participants that are asked to evaluate the spatial extent of growing lines is not influenced by their duration^23^.

In addition, several studies have also reported a strong interplay between the perception of time and numerosity. Studies on animal models suggest that time and numerosity might be represented via a shared representational mechanism in which an ‘internal accumulator’ represents the numerosity or duration of events/objects by summing up the impulses yielded by a generator^24^. This common code for time and numerosity seems to be supported by studies showing that in rats and both infants and newborn humans, a numerical rule can be generalized into the temporal domain and vice versa^24–26^. These findings are complemented by psychophysical evidence showing that in human adults, the numerosity of visual stimuli interferes with the judgement of their duration^27^. Nonetheless, it is worth noting that robust asymmetric effects have also been reported for the interaction between time and numerosity: when participants judge the numerosity, the duration of the stimuli does not significantly affect numerosity estimates^27^. In addition, emotional stimuli affect time and number in opposite ways, as these have been reported to yield overestimating temporal estimates but *under*estimation of numerical estimates^28^.

Even more critical for the idea of a shared magnitude mechanism are reports documenting asymmetric or conflicting effects for the different dimensions since the very first stages of life. For example, it has been measured the ability of 4-months old infants to detect ordinal relationships between magnitude sequences. At a first glance, magnitudes appear to share the same pattern of development, with infants being able to successfully discriminate between series of stimuli ordered in increasing magnitude, when the manipulated variable is size^29^, non-symbolic numerosity^30^ or duration^31^; all evidence in line with the idea of a magnitude shared system. However, there are some key differences among the domains, with discrimination acuity being lower for numerosity compared to size^30^ and infants being unable to discriminate between sequences with decreasing magnitude in size^29^ and numerosity^30^ while they turned out to be able to do so in the temporal domain^31^. These findings suggest that ordering operations share a similar but not completely overlapping path of development, thus casting doubts on the idea of a common magnitude system. Also experiments concerning adult participants reported contrasting evidence. For example, in two recent studies, space, time and number were pitted against each other, and participants were asked to judge the duration, the numerosity or the spatial extent of visual stimuli. One of these studies reported a significant interference of numerical and spatial information on temporal judgments^32^, while in the other study temporal judgments were not affected by space or number, even though the opposite was true^33^. In addition, some studies failed to find any cross-dimensional interference. For instance, in an estimation task of auditory signals, perceived numerosity was not found to affect temporal estimates^34^ while, in another experiment, no interference between numerosity and duration was reported in high working-memory load tasks^35^. It is crucial to highlight that all the studies mentioned above used a different task to investigate a cross-magnitude interference. In particular, Dormal and Pesenti^32^ used a comparison task, in which participants had to indicate which stimulus lasted longer. This study found a positive interference of numerosity on duration similarly to Xuan et al.^20^ that, interestingly also employed a comparison task. However, not all studies using a comparison task reported interaction. For example, Bi et al.^35^ reported that changes in working memory load did not trigger any significant interference between numerosity and duration. Finally, the null effect found by Agrillo et al.^34^ might be accounted for in two different ways. First, in that experiment stimuli belonged to the auditory modality that has a higher temporal resolution compared to vision^36^, which might prevent cross-magnitude interference from happening. Second the exploited task: an estimation task, which does not require an explicit comparison between two stimuli and relies on visuomotor response.

Here we hypothesize that the current empirical discrepancies could be related to crucial methodological differences among the studies, and that specific experimental paradigms might be more prone to induce cross-dimensional interference effects. This idea finds support in the results of a study by Yates et al.^37^. These authors found that in a discrimination task similar to that used by Xuan^20^, the perceived size of visual stimuli significantly affected temporal judgments, so that larger stimuli were estimated as lasting longer, thus replicating previous results. However, when participants were required to judge whether the same stimuli had the “same or different” duration, thus eliciting a direct semantic instantiation of the concept of “magnitude”, the opposite effect was found, with larger stimuli being judged as lasting shorter.

The main goal of the present study is to test whether, in line with recent evidence about the study of the space-number association^38,39^, the use of magnitude-related response codes, e.g. “shorter” vs “longer” plays a crucial role in triggering and guiding the functional interaction between time and numerosity perception, in a way similar to that documented by Yates et al.^37^ for the interaction between size and duration. In four different experiments, we asked participants to discriminate or estimate the duration of visual stimuli (dots arrays) while manipulating the numerosity of the set, despite this information being completely irrelevant to the task. In a discrimination task, participants indicated which one out of two sequentially presented stimuli had the longer duration by indicating its position in the sequence (first or second; Experiment 1 “Discrimination task”). In Experiment 2 (“Equality task”), the same stimuli were used, in order to control for any perceptual difference, however the task was changed: participants provided equality judgments by indicating whether the two sequential stimuli had the same or different duration. To test the role of decision making, a task in which participants did not have to choose between two stimuli was devised: on each trial the temporal duration of dot arrays had to be reproduced via key press (Experiment 3, “Reproduction Task”). Finally, an additional control experiment was designed to rule out any possible effect of ordering (as could have been triggered by having to indicate the first or second stimulus in order of appearance). For this reason, Experiment 4 (“Discrimination for colored stimuli”) was a direct replication of Experiment 1, with the only difference that stimuli were colored (red and blue vs green and yellow) and participants had to indicate which one lasted longer by reporting the color of the longer stimulus, not its relative position in the series.

If the perception of duration and numerosity relies on basic bottom-up shared neural/functional mechanisms, we expect the interaction between these two dimensions—in the form of biases induced by stimulus numerosity on duration estimates—to occur in all experiments. In contrast, if the interplay between the perception of duration and numerosity is guided top-down by the type of task participants are engaged in, and the use of magnitude-related response codes like “shorter” vs “longer”, equality judgements and reproduction tasks would be likely to reveal inconsistencies in the cross-dimensional interactions. If this were the case, one should conclude that cross-dimensional interactions do not arise because the magnitude of time and numerosity dimensions is processed together at the perceptual level and automatically coded in a bottom-up fashion by a shared magnitude system. Rather, cross-dimensional interferences would be induced “top-down” by task-related processes such as the way sensory inputs are classified through response codes, represented in short-term memory and matched against each other for decision making. The series of experiments presented here reveals a highly specific interaction between time and numerosity only in discrimination tasks where the use of contrasting “shorter” vs “longer” responses provide a top-down magnitude coding bias. These results suggest that the occurrence of cross-dimensional interferences is task-independent and that, therefore, it is imprudent to generalize the functional and theoretical implications of these interferences without considering the task conditions at the origin of these interferences.

## Results

### Experiment 1: discrimination task

**Figure 1:**
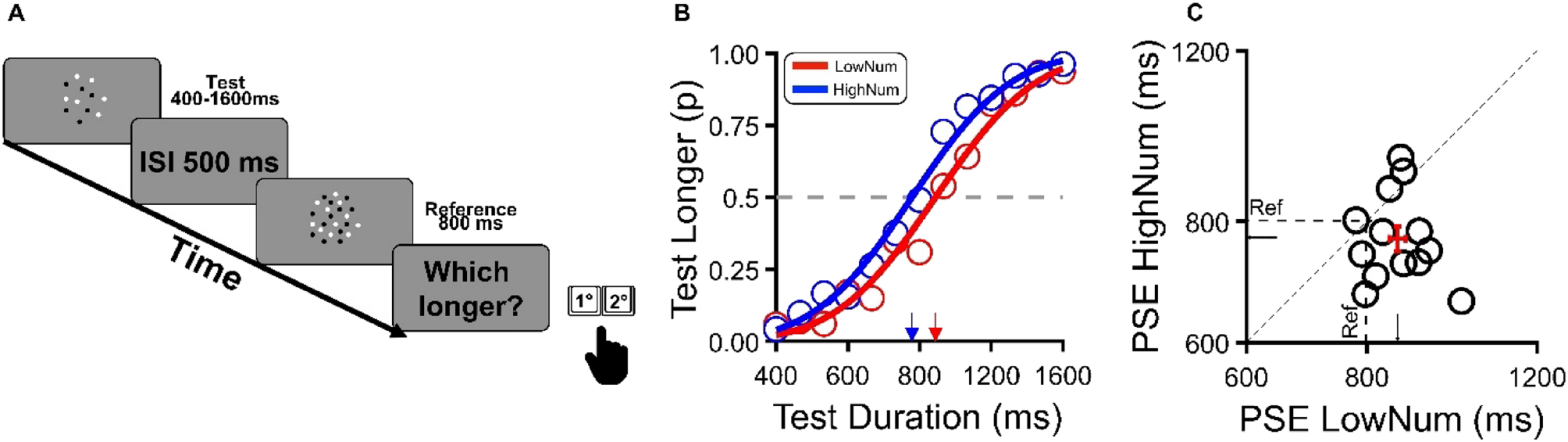
Discrimination task. (**A**) Participants were asked to indicate which of the two clouds of dots presented sequentially lasted longer. In all trials, the reference stimulus consisted of 24 dots and had a fixed duration of 800 ms. The test stimulus had a variable duration, between 400 to 1600 ms, and its numerosity was either 12 (low numerosity) or 48 (high numerosity), with the two conditions tested in separate sessions. (**B**) Aggregate data about the proportion of “test longer” responses plotted against test duration. The 50% point of the best-fitting cumulative Gaussian curves indicates the PSEs, that is, the physical duration of the test stimulus to perceptually match a reference of 800 ms. Conditions with different test numerosity are indicated by color: low numerosity (N = 12) in red and high numerosity (N = 48) in blue, respectively. (**C**) Individual PSEs (open circle) for each participant in the low numerosity condition (x-axis) plotted against the high numerosity condition (y-axis). Dots falling below the bisection line (dashed, diagonal line) indicate a higher PSE in the low numerosity condition compared to the high numerosity condition, and thus a positive covariation between perceived numerosity and duration with more numerous stimuli perceived as lasting longer than their physical duration (and vice-versa). Filled circles indicate the PSE ± S.E.M.

Participants were required to discriminate the duration of two clouds of dots. In each trial, the duration of the reference (randomly presented as first or second) was fixed and equal to 800 ms, while the duration of the test varied according to an adaptative QUEST algorithm^40^ capped between ± 0.3 log units (Fig. 1A). We designed two experimental conditions (tested in separate sessions): in the “low numerosity” condition, the numerosity of the test was half of that of the reference (12 vs 24), while in the “high numerosity” condition it was twice as much (48 vs 24). Figure 1B shows the results of the aggregate data obtained from all participants. The proportion of “test longer” responses was plotted as a function of test duration for both the low and high test numerosity conditions. The 50% point of the Gaussian distribution represents the point of subjective equality (PSE), that is, the physical duration of the test to be perceptually matched to the duration of the reference. A leftward shift of the curve relative to reference duration indicates a bias to overestimate the duration of the test relative to an ideal observer, while a rightward shift indicates underestimation. More importantly, the relative position of the curves for the high and low numerosity conditions provides a quantitative estimate of the distortions induced by stimulus numerosity on time perception. When the test stimulus was more numerous than the reference (N 48 vs 24), its perceived duration was overestimated (PSE = 771 ms) relative to the condition in which the test numerosity was lower than the reference (N 12 vs 24, PSE = 864 ms). It is worth noting that this 100 ms difference between the high and low experimental condition was induced by the relative difference in numerosity between the test and the reference despite such dimension being irrelevant to the accomplishment of the temporal task. Figure 1C shows individual data with the PSEs in the low numerosity condition plotted against the PSEs in the high numerosity condition. It is clear from inspection that, despite a consistent variability, for most participants, PSEs in the low numerosity condition were larger than in the high numerosity condition, indicating that stimuli of larger numerosity were perceived to last longer. To statistically test for this difference, individual PSEs were used in frequentist and Bayesian paired sample *t*-tests. The frequentist analysis revealed a significant difference between high and low numerosity conditions (t(12) = 3.002, p = 0.01, d = 0.83) in line with the Bayesian analysis (Bf10 = 5.35), indicating substantial evidence in favor of H1 (interference between the perception of duration and numerosity).

### Experiment 2: equality task

**Figure 2:**
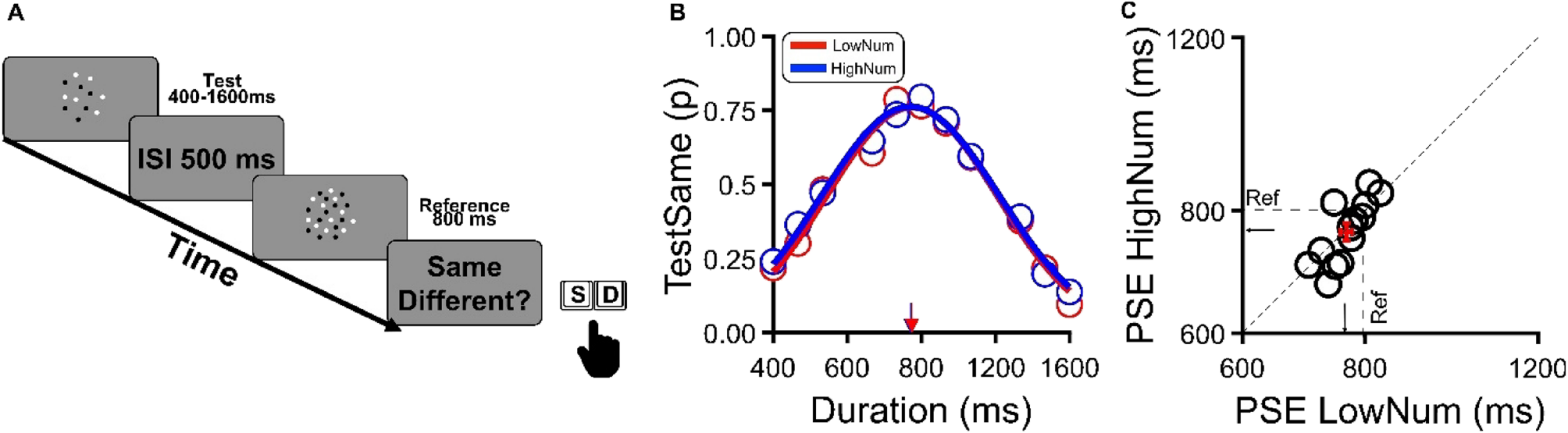
Equality task. (**A**) Participants were asked to indicate if two stimuli presented sequentially had the same or different duration. All stimuli, test (variable) and reference (fixed) were identical as in Exp 1. (**B**) Results for the aggregate data. The probability of perceiving the test stimulus as lasting the same as the reference (800 ms) plotted against several test durations for the low numerosity condition (N test = 12) and high numerosity condition (N test = 48) in red and blue, respectively. Continuous colored curves indicate the best-fitting Gaussian to the data. The peaks of the Gaussians (indicated by arrows on the x-axis) indicate the PSEs, that is, the physical test duration needed to perceptually match the reference (800 ms). (**C**) Individual PSEs (open dots) for each participant in the high numerosity condition plotted against PSEs for the low numerosity condition. The central cross represents the average perceived duration and error bars ± S.E.M.

In Experiment 2, participants were asked to judge whether two stimuli in a sequence had the same or different duration. As with Experiment 1, we designed two experimental conditions: in the “low numerosity” condition, the variable (test) stimulus was 12 (half of the reference), in the “high numerosity” condition, 48, twice the reference (see Fig. 2A). Figure 2B,C show the results for the aggregate data averaged across all participants with the proportion of “same” responses plotted against the test duration. The mean of the best-fitting Gaussian curve in Fig. 2B indicates the physical duration of the test to be perceived as being identical to the reference (800 ms). In both experimental conditions, the peak of the Gaussian curves was found to be very close to 800 ms and indeed rather similar to each other (765 ms and 760 ms for the high and low test numerosity conditions, respectively), suggesting that, when engaged in equality not discrimination tasks, observers were able to encode visual stimulus duration regardless of their numerosity. In other words, the processing of stimuli duration and numerosity are independent of each other in equality judgements, with no sign of any cross-dimensional interference effect. Such a claim was fully supported by both frequentist and Bayesian paired samples *t*-tests. The former revealed that the difference between the two experimental conditions was far from significant (t(12) = 0.48, p = 0.64, d = 0.13), and the latter indicated evidence supporting H0 (Bf10 = 0.308).

### Experiment 3: reproduction task

**Figure 3:**
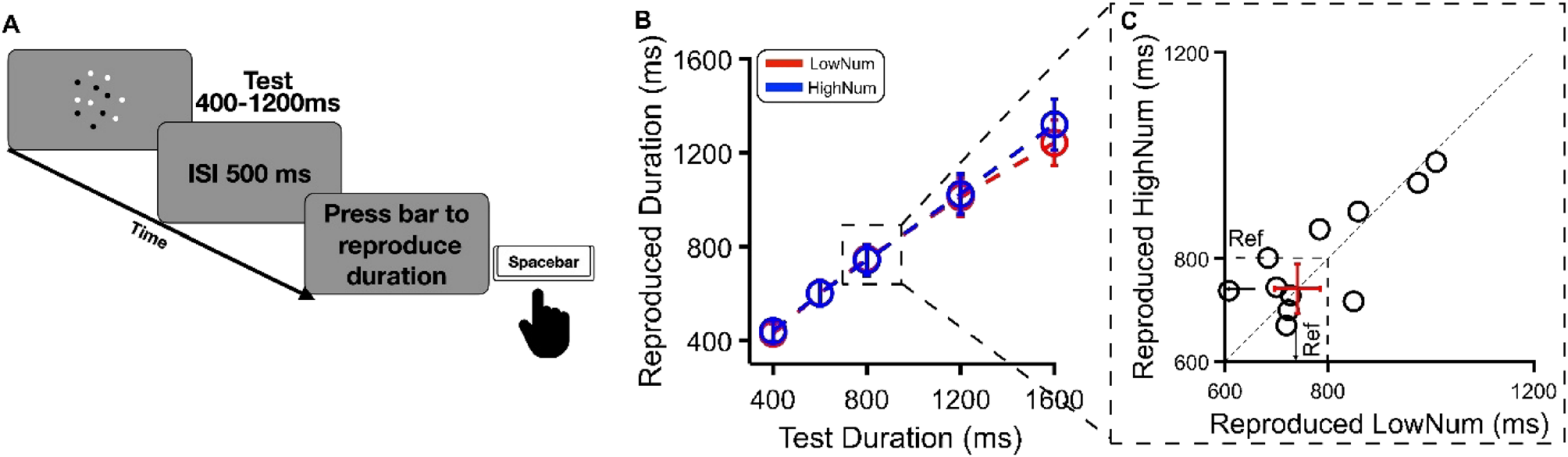
Reproduction task. (**A**) Participants were asked to press a key on the keyboard to reproduce the duration of a cloud of dots displayed at the center of the monitor. As in Exp 1 and 2, two different experimental conditions were designed: in the high numerosity condition, the test stimulus comprised 48 dots, while in the low numerosity condition, numerosity was equal to 12 (tested in separate sessions). (**B**) Results for aggregate data. Reproduced test durations are plotted as a function of physical stimulus duration with the low (N12) numerosity condition and the high numerosity (N48) condition colored in red and blue, respectively. Open symbols represent the average reproduced duration for each tested interval ± S.E.M. (**C**) Reproduced duration for the 800 ms interval for each participant in the low plotted against the high numerosity condition (open symbols). The central cross indicates the average perceived duration across all participants ± S.E.M.

In this experiment, participants were asked to estimate the duration of a visual array containing either 12 or 48 dots displayed on a monitor screen and then reproduce it as accurately as possible by holding down a response key (Fig. 3A). The averaged reproduced intervals for the 5 tested durations (400, 600, 800, 1200 and 1600 ms) are shown in Fig. 3B for the low (N12) and high (N48) numerosity conditions. For each subject and tested duration, reproduced durations falling beyond 3.2 standard deviations from the mean were excluded from further analyses. As it is clear from inspection, duration reproduction was similar between the high and low numerosities (429, 602, 749, 1009 and 1241 ms for low and 440, 601, 742, 1023 and 1320 ms for high numerosities, respectively) with no difference between high or low test numerosity. The reproduction data for the 800 ms is of particular interest as it matched the reference duration in Experiments 1 and 2. Figure 3C shows individual data for time reproduction in the low numerosity against the high numerosity for 800 ms. Almost all data points are scattered along the diagonal (veridical line), suggesting that the reproduction of the observed interval was not significantly affected by stimulus numerosity, a pattern of results which also generalized to the other tested durations (those shorter or longer than 800 ms). To quantify the effects, we ran a two-way repeated measures ANOVA with test numerosity (low or high) and 6 durations (400, 600, 800, 1200 or 1600 ms) as factors. The results revealed a trivial significant effect of duration (F(1.53, 18.36) = 302.76, p < 0.001, ηp^2^ = 0.96), indicating that participants' responses covaried with interval magnitude with longer durations being reproduced as lasting longer and vice versa. Importantly, the factor numerosity (F(1,12) = 0.11, p = 0.75, ηp^2^ = 0.009) and the interaction between duration and numerosity (F(2.83, 33.92) = 0.61, p = 0.60, ηp^2^ = 0.049) were not statistically significant. A Bayesian analysis, collapsing the data for the five different durations, nicely complemented these results suggesting evidence for H0 (Bf10 = 0.29).

### Experiment 4: discrimination task for colored stimuli

**Figure 4:**
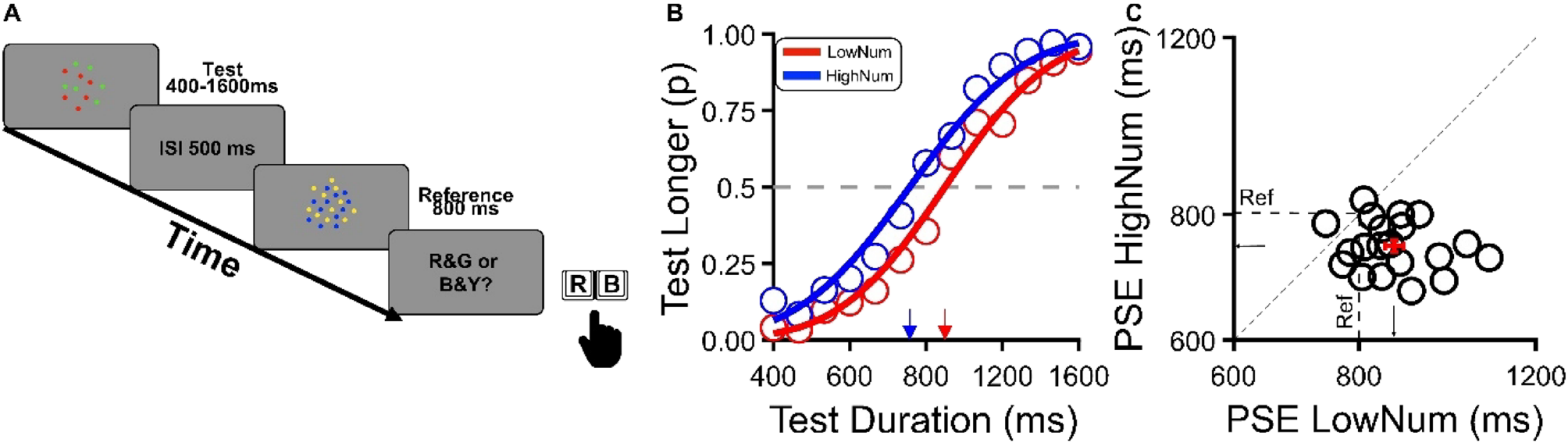
Discrimination Task for colored stimuli. (**A**) Duration discrimination with colored stimuli. All procedures were identical to the discrimination task of Exp. 1, but the test and reference stimuli were colored differently: red/green or yellow/blue (color assignment counterbalanced across trials). In each trial, participants were required to indicate the color (not the position in the sequence) of the stimulus that lasted longer. (**B**) Aggregate data. The proportion of “test longer” responses plotted against test duration (open symbols) and best-fitting cumulative Gaussian functions to the data (solid lines). The 50% point of the psychometric function (indicated by arrows) represents the PSE. The rightward shift of the curve for the low numerosity condition (red) relative to the high numerosity condition (blue) indicates an interference effect of stimulus numerosity on duration estimates, with more numerous stimuli perceived to last longer and vice versa. (**C**) Individual PSEs in the low numerosity condition (N = 12) plotted against those measured in the high numerosity condition (N = 48). Almost all data points are displaced below the diagonal line, indicating that PSEs in the low numerosity were longer than in the high numerosity condition and thus, that less numerous stimuli were perceived to last shorter and vice versa.

The main result of the series of tests presented above is that perception of time and numerosity interacts only in the duration discrimination experiment and they were found to be independent in the equality (same/different) and reproduction tasks. Given that the very same intervals and visual stimuli were exploited in all experiments, it is likely that the selective effect reported in time discrimination is related to the procedures followed to accomplish this task. Indeed, in the duration discrimination experiment, observers were required to indicate which stimulus, whether the first or second lasted longer with this ordering code that might have induced the exploitation of all available quantitative information, and thus even the difference in numerosity between the test and the reference. To test for this hypothesis, we devised a new version of Experiment 1 in which test and reference stimuli were colored differently and participants did not indicate the position of the longest stimulus in the sequence but just its color. The test and reference either consisted of a cloud of red and green dots or, alternatively, blue and yellow dots with the colors of the two stimuli counterbalanced across trials. Figure 4 shows the results of the aggregate data across all participants. As in Fig. 1B, the proportion of “test longer” responses are plotted against test duration with the low and high numerosity conditions (test numerosity one-half or twice the reference) displayed as separated curves. The results indicate that when the test was more numerous than the reference, its duration was overestimated, as indicated by a PSE of 781 ms. However, the opposite held true for the low numerosity condition with a PSE achieved for a test physical duration of about 840 ms. Again this was confirmed by both a frequentist paired-sample *t*-test (t(19) = 5.66, p < 0.001, d = 0.46) and a Bayesian approach (Bf10 = 1262). This result perfectly matched the result of Experiment 1, supporting the idea that, in discrimination tasks, an interplay between perceived numerosity and duration occurs independently the response code used by the participants.

### Duration biases as a function of tasks: an overview

**Figure 5:**
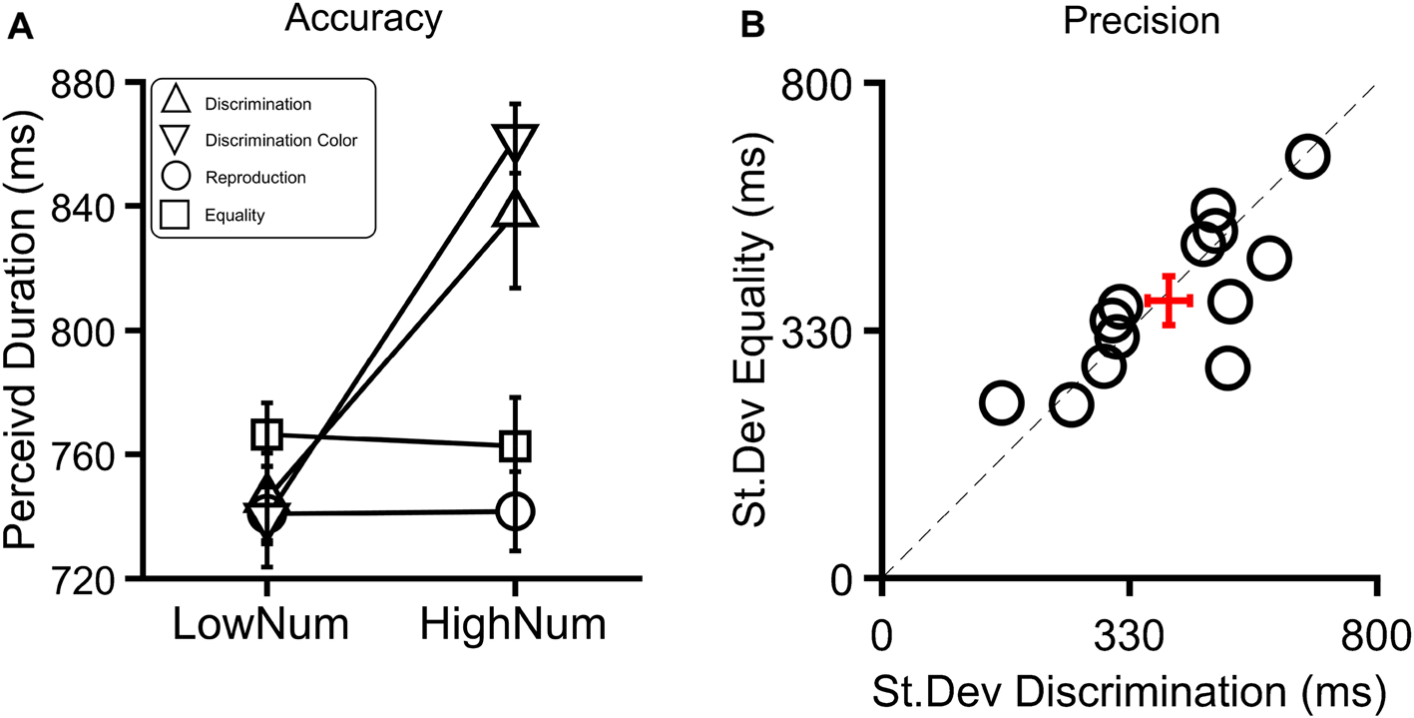
Accuracy and precision. (**A**) Matching duration estimates across the 4 experiments. Test stimuli estimate to match an 800 ms interval in the time discrimination, equality and reproduction task for the condition in which test numerosity was 12 or 48, low and high numerosity conditions, respectively. While in the equality (same/different) and reproduction task, test duration estimates were found to be independent of the test numerosity (squares and upward triangles) in both discrimination tasks, perceived duration and numerosity covaried to each other with more numerous stimuli perceived to last longer and vice versa (circles and down triangles). (**B**) Responses precision in the four experiments. We indexed the precision of participants' responses in the discrimination and equality task via the standard deviation of the response distributions. On average, SD values were quite similar for both tasks, those in which perceived numerosity interfered with duration estimates (discrimination task, Experiment 1) as well as those in which no signature of interference was observed (equality task, Experiment 2).

To better achieve a quantitative estimation of the interference between the perception of stimuli duration and numerosity in the four different tasks, we measured for each of them the perceived or reproduced duration matching an interval of 800 ms as a function of stimulus numerosity (high and low). The results shown in Fig. 5A indicate a clear interaction between duration biases induced by numerosity and tasks. A 3 × 2 ANOVA with task (Discrimination, Equality and Reproduction) and numerosity (high or low numerosity) as factors confirmed the interaction (F(2,24) = 8.34, p = 0.002, ηp^2^ = 0.41), indicating that the effect of numerosity on duration perception was modulated by the task. A series of post-hoc *t*-tests confirmed that the only significant effect of numerosity on time was elicited by discrimination tasks employed in Experiments 1 and 4 regardless of participants indicating the longer stimulus by reporting its position in the sequence (ordering information, Experiment 1, t = 3.97, p = 0.006, Bonferroni corrected, d = 0.68) or its color (Experiment 4, t = 5.66.1, p = 0.001, Bonferroni corrected, d = 0.46). In both discrimination tasks, the perceived duration of the test in the high numerosity condition (N = 48) was about 100 ms longer than in the low numerosity condition (test numerosity half the reference, N = 12). In contrast, neither in the equality (same/different) nor in the duration reproduction task stimuli numerosity affected duration estimates. In the equality task, the test was matched to an 800 ms reference when its physical duration was slightly shorter (40–50 ms), indicating a small bias to overestimate the duration of the variable stimulus. Similarly, participants, on average, reproduced a visual stimulus lasting 800 ms by demarking an interval of about 740 ms. More importantly, a change of a factor of 4 in test numerosity (from 12 to 48) did not significantly affect duration estimates, neither in the equality nor in the reproduction tasks.

### Sensory precision

One possibility to account for the task-dependent interaction between perceived numerosity and duration might be the differences in difficulty between the tasks. For example, discriminating between the duration of two stimuli might have been more difficult than categorizing them as identical or different, and this, in turn, might have automatically prompted the observers to leverage on all available quantity information and contaminate duration estimates with numerosity information. To test for the hypothesis that cross-dimensional interactions (i.e. time and numerosity) depend on task difficulties, we measured averaged response precision for the purely perceptual tasks (discrimination and equality) under the plain assumption that the more difficult the task, the lower the average response precision. In doing so, we retrieved the standard deviation (SD) of the best-fitting psychometric curves, plotting the probability of perceiving the test stimulus as longer against its physical duration in Experiment 1. Then, we measured the SDs of the best-fitting Gaussian curves in Experiment 2, plotting the probability of the same responses against the relative duration of the test and reference. Before contrasting the values across the two experiments, we first checked whether, in any of them, the SDs differed between the high and low numerosity conditions. As no statistically significant difference was observed (both p-values > 0.2), we averaged SD values between the high and low numerosity to obtain a single estimate for each experiment. As shown in Fig. 5B, despite a rather large variability between participants, the average SDs were similar in the two experiments (336 ms and 322 ms, respectively). The lack of difference between participants’ response precision—which we used to index task difficulty—was also supported by a paired-sample *t*-test (t(12) = 0.6, p = 0.53, d = 0.17). In other words, despite the fact that perceived numerosity and duration interacted in the discrimination but not the equality (same/different) task, the difficulty of the two tasks turned out to be rather similar, suggesting that complexity is not a critical factor in defining the interference effects between the two perceptual dimensions.

## Discussion

In the current study, we tested whether numerosity affects time perception. The numerosity–time interaction was measured with several different tasks and judgment procedures while using the same stimuli and tasks matched for difficulty. Across four experiments, duration perception was measured against discrimination, equality judgments, and reproduction tasks. The results revealed a surprisingly task-specific interaction, with only discrimination tasks inducing a bias in temporal duration judgements as a function of stimuli numerosity, with more numerous stimuli being perceived as lasting longer. While behavioral magnitude interactions have been classically interpreted as a key proof for the existence of a generalized magnitude system, the current results question this as it highlights the need to carefully control for task-related factors (such as susceptibility to decisional biases) before drawing general conclusions. It is worth mentioning that this is not the first study highlighting a similar, controversial issue. Xuan et al.^20^ asked participants to judge which of two visual squares lasted longer (discrimination task). The results showed that relatively bigger squares were perceived as lasting longer compared to smaller squares, an effect also found by leveraging on a duration reproduction paradigm^22^. However, Yates et al.^37^ measured the effect of size on time perception by employing two different tasks. The rationale was that if size genuinely affects time perception, the interference between these two dimensions, indexed in terms of bias in duration judgements, should occur independently of the task. The results obtained with a discrimination task similar to that used by Xuan et al.^20^ replicated the main effect, with bigger squares being judged as lasting longer. Crucially, when the effect was measured via an equality task, the results showed an opposite timing bias, with larger stimuli perceived to last shorter. The authors concluded that task-dependent decisional biases play a key role in generating cross-dimensional interactions in magnitude judgements. Also the well-investigated SNARC effect^41^ seems to be modulated by contextual effects and, more importantly, by the task employed, thus suggesting a top-down process. For instance, the direction of the SNARC effect reversed after participants were asked to image numbers on a face-clock instead of a ruler^42^. More relevant to the present findings, Mingolo et al.^43^ have recently demonstrated how eliciting a new spatial organization of numbers (namely the phone key-board configuration) can modulated the SNARC effect. However, this modulation depended on the task participants are engaged into, with a number magnitude task eliciting no SNARC effect after being exposed to the phone key-board configuration while a canonical SNARC effect was found when participants were asked to perform number parity judgments. These examples imply the need for careful experimental monitoring of how decisional factors can influence the results of investigations focused on cross-dimensional magnitude processing and the interpretation of the same results.

Before the present study, whether the perception of size and duration is the only cross-dimensional interplay to show a task-dependent selectivity or whether it generalizes to other domains was an unanswered question. Two previous studies, that employed a discrimination task where participants had to judge which stimulus lasted longer, reported a significant interaction between number and time magnitudes^20,44^. The method used in those studies is equivalent to the one employed here in Experiments 1 and 4, which, notably, were the only two experiments in which we found a significant influence of numerosity on time. Importantly, some previous studies failed to detect a significant interaction between the perception of numerosity and time durations^45–47^. However, it is worth noting that these studies used Arabic digits rather than visual numerosity stimuli. Recent fMRI investigations have demonstrated that the neural correlates of numerosity discrimination of symbolic and non-symbolic number stimuli activate networks of brain areas that are not entirely overlapping^48^. This result suggests that the interaction between numerosity and duration might be selective for the type of numerosity format considered in a study.

One possible explanation for our results is that discrimination judgments are more difficult compared to other tasks, and this, in turn, would make discrimination judgments more prone to the influence of the irrelevant numerosity magnitude. However, in our study, no significant difference in sensory precision was found between the discrimination (Experiment 1) and the equality task (Experiment 2). This result rules out the possibility that the numerosity/time interaction found in Experiment 1 and 4 can be accounted for by higher task difficulty.

Another possibility is that the bias induced by numerosity on duration perception occurs at the decisional level. The peculiarity of the discrimination task is that is the only forced-choice paradigm. In these tasks the criterion is completely eliminated, whilst it plays a critical role in the “non-forced choice” tasks in which participants are free to set a threshold to make their decisions. Being pressed to make a decision might have induced the observers to use all available information, even if this was task-irrelevant, thus triggering the interaction between time and numerosity. Although fascinating, this hypothesis cannot be tested with the present data thus, future studies have to tackle this issue directly.

In their original paper, Yates et al.^37^ aimed at determining whether larger stimuli are judged to last longer because size affects perceived duration or because size biases decisions about duration. Here we wish to develop this suggestion and propose that our data shows that response codes used for judging the numerosity magnitude also bias decisions on the magnitude in the time-duration domain. Yates et al.^37^ noted that in magnitude-interaction paradigms, decision effects could be guided by “strong conceptual and linguistic similarities between magnitude across different dimensions”. Here we argue that when a participant is asked to use contrasting and dichotomic decision codes, a shared magnitude representation across different dimensions is triggered because these very codes can be used to express and communicate magnitude across different dimensions. This, in turn, would activate a semantic representation of magnitude that is superordinate to these different dimensions. In contrast, “qualitative” response codes like “same or different” are not directly or exclusively related to the concept of magnitude, thus, might not activate a semantic representation of magnitude that is superordinated to space, number and time.

To summarize, our results suggest that the functional interaction among magnitudes from the different domains of space, number and time might not be guided by a bottom-up sensory mechanism or by a shared bottom-up coding of magnitude. Rather, the results of our experiments suggest that high-level and top-down processes involved in decision-making and guided using “magnitude-related” response codes play a relevant role in generating interactions and interferences during the coding of magnitude in space, number and time domains (see^38,39^). To disentangle between the “bottom-up” vs “top-down” origin of interference effects among these domains, future studies should be designed to include more than a single perceptual task.

To conclude, while a task-dependent interaction between numerosity and time does not rule out, “per se”, the possibility of the existence of a system dedicated to the coding of magnitude across these dimensions, it is also true that evidence for interference effects in the perception of different perceptual dimensions does not straightforwardly imply that these interferences originate from an automatic, bottom-up and shared common mechanism that codes magnitude across these dimensions.

## Methods

### Stimuli

All experiments were carried out in a dimly lit and sound-attenuated room with stimuli presented on the monitor of an iMac (5120 × 2880 resolution, refresh rate 60 Hz) subtending 60° × 34° at the subject view distance of 57 cm. Visual stimuli were created via the PsychToolbox routine for MatLab (v. R2016b, Mathworks, Inc.) and consisted of clouds of dots (each element had a diameter of 0.2°) inscribed in an invisible circular area of 12° diameter centered on the monitor screen. In all conditions except for Experiment 4, the dots in the set were half white and half black to keep the mean luminance identical to the mid-grey background. In experiment 4, the two clouds of dots in the sequence were colored differently, blue/yellow or red/green. The request for the participants was to indicate the color (not the position in the sequence as in Exp 1) of the stimulus lasting longer.

### Subjects

A total of 23 participants took part in the experiments (mean age 28.07 ± 7.2, 9 males). Thirteen of them participated in Experiments 1, 2 and 3; 20 participated in Experiment 4 with a subgroup of 8 participants that participated in all experiments. All observers had normal or corrected-to-normal vision and provided written informed consent before starting the experiments. The experimental procedures were approved by the local ethic committee (“Commissione per l’Etica della Ricerca”, University of Florence, 7 July 2020, n. 111) and by the Ethics Committee of the Fondazione Santa Lucia IRCCS (Date 12/12/2020 / No. CE/PROG.895). All the experiments were conducted in accordance with the Declaration of Helsinki.

### Experiment 1—discrimination task

In a two-alternative forced choice (2AFC) task, participants were asked to indicate which of two sequentially presented clouds of dots (500 ms of ISI) had a longer duration. Participants were required to press the “a-key” to indicate the first and the “s-key” to indicate the second stimulus in the sequence (Fig. 1A). In all trials the reference stimulus contained 24 dots and had a duration of 800 ms. The duration of the test stimulus varied from trial to trial and was selected according to an adaptive staircase QUEST^40^ with the range of duration constrained between 400 and 1600 ms. Two different experimental conditions, measured in separate sessions, were defined by the numerosity of the test: low numerosity condition (12 dots) and high numerosity condition (48 dots). After the presentation of the two visual stimuli, a color change of the central fixation point prompted the participants to provide a response, then, after a pause of 500 ms, a new trial automatically started. The presentation order of test and reference was randomized across trials while the order of experimental conditions (defined by test numerosity) was counterbalanced across participants. For each subject, in each condition, we collected 3 sessions of 30 trials, for a total of 90 trials per condition.

### Experiment 2—equality task

Participants were required to compare two visual stimuli presented sequentially and indicate whether their duration was identical (response “same”, to be indicated by pressing the “a-key”) or not (response “different”, ”s-key”; see Fig. 2A) by using a computer keyboard. Stimuli and procedure were the same as in Experiment 1, with a reference stimulus containing 24 dots and having a duration of 800 ms. The only difference was the sampling procedure of the test duration, which was defined by a logarithmically spaced distribution ranging from 400 to 1600 ms in eleven steps rather than by an adaptative staircase. Similarly to Experiment 1, the presentation order of test and reference was randomized across trials, and the two experimental conditions defined by high or low test numerosity were presented in separate sessions. For each subject, in each condition, we collected 3 sessions of 66 trials (each duration randomly presented 3 times), for a total of 198 trials per condition.

### Experiment 3—reproduction task

In this experiment, participants were required to reproduce the duration of a visual stimulus (a cloud of dots) by holding down the spacebar on a computer keyboard (Fig. 3A). As in Exp 1 and 2, test stimulus numerosity was either 12 or 48 dots (tested in separate sessions), and the test duration ranged between 400 and 1600 ms demarking 5 different intervals: 400, 600, 800, 1200 and 1600 ms. Participants were required to reproduce the perceived interval as accurately as possible soon after the offset of the visual stimulus. No time constraint was applied to interval reproduction.

### Experiment 4—discrimination task for colored stimuli

Experiment 4 was identical to Experiment 1, but the visual stimuli were colored, not achromatic. On each trial, the dots of one cloud (randomly test or reference and first or second in the sequence) were 50% red and 50% green, while those of the other were 50% yellow and 50% blue (Fig. 4A). Participants were instructed to indicate the color of the stimulus that lasted longer by responding with a key press. Note that this procedure required identifying the more numerous stimuli via a color label and not by its position in the sequence. This controlled for the possibility that cross-dimensional interferences reported in Experiment 1 were prompted by the ordering information exploited to accomplish the task.

### Data analysis

For Experiments 1 and 4 (both Discrimination Tasks), the proportion of trials in which the test appeared as “longer” than the reference was plotted against the test duration and fitted with a cumulative Gaussian error function separately for the two test numerosity conditions (high and low). The 50% point of the error functions indicates the point of subjective equality (PSE). The width of the Gaussian function (standard deviation) was taken as an index of sensory precision (see Fig. 2A).

In Experiment 2 (Equality Task) perceptual accuracy was measured by plotting the proportion of trials in which the test was judged as having the same duration of the reference as a function of the test stimulus’ physical duration (examples in Fig. 3A). Data were then fitted with Gaussian functions, with the peak indicating that the physical duration of the test stimulus was perceptually matched to the reference (PSE). The standard deviation of the best-fitting function was instead taken as an index of perceptual precision.

In Experiment 3 (Reproduction Task), for each duration, we calculated the mean and the standard deviation of reproduced duration for each test duration, indicating observers’ accuracy and precision, respectively. For each participant before computing the final average, reproduced durations falling beyond 3.2 standard deviations from the mean of the responses for that duration was excluded from further analysis. This led to the rejection of less than 2% of total trials. Statistical significance was tested via frequentist repeated measures ANOVAs and *t*-tests; whenever the sphericity assumption was violated, the Greenhouse–Geisser correction was applied. We also performed repeated measures Bayes-factors *t*-test along with the frequentist *t*-test. A BF10 = 1 indicates no evidence for either hypothesis, while a BF10 higher than 1 suggests evidence for the alternative hypothesis, with the robustness of the evidence increasing as the BF10 increases. Evidence of H0, on the other hand, is suggested with a BF10 lower than 1, in this case, a lower BF10 indicates stronger evidence for H0. All statistical analyses were performed with MatLab 2016b (The Mathworks, Inc., Natick, MA, USA) and Jasp Software (version 0.14.1; JASP Team, Amsterdam, The Netherlands).

## Data Availability

Data for all the experiments are available at: https://zenodo.org/record/8024907.
